# Structurally reconfigurable designer RNA structures for nanomachines

**DOI:** 10.52601/bpr.2021.200053

**Published:** 2021-02-28

**Authors:** Kai Jiao, Yaya Hao, Fei Wang, Lihua Wang, Chunhai Fan, Jiang Li

**Affiliations:** 1 Division of Physical Biology, CAS Key Laboratory of Interfacial Physics and Technology, Shanghai Institute of Applied Physics, Chinese Academy of Sciences, Shanghai 201800, China; University of Chinese Academy of Sciences, Beijing 100049, China; 2 School of Chemistry and Chemical Engineering, Frontiers Science Center for Transformative Molecules and National Center for Translational Medicine, Shanghai Jiao Tong University, Shanghai 200240, China; 3 The Interdisciplinary Research Center, Shanghai Synchrotron Radiation Facility, Zhangjiang Laboratory, Shanghai Advanced Research Institute, Chinese Academy of Sciences, Shanghai 201210, China

**Keywords:** RNA nanomachine, Structure, Reconfiguration, Biocomputing, Theranostics

## Abstract

Structurally reconfigurable RNA structures enable dynamic transitions of the functional states in response to diverse molecular stimuli, which are fundamental in genetic and epigenetic regulations. Inspired by nature, rationally designed RNA structures with responsively reconfigurable motifs have been developed to serve as switchable components for building engineered nanomachines, which hold promise in synthetic biological applications. In this review, we summarize recent progress in the design, synthesis, and integration of engineered reconfigurable RNA structures for nanomachines. We highlight recent examples of their targeted applications such as biocomputing and smart theranostics. We also discuss their advantages, challenges as well as possible solutions. We further provide an outlook of their potential in future synthetic biology.

## INTRODUCTION

Nucleic acid base-pairing and protein–nucleic acid interactions in living systems are precisely encoded by nucleic acid sequences, which enable complex biomolecular networks and lay the foundation of synthetic biology (Ma *et al*. 2012; Purnick and Weiss 2009). In recent decades, great efforts have been made to engineer biological structures and nanomachines for diverse applications, such as biocomputing, nanofabrication and smart theranostics (Ge *et al*. 2018; Hu *et al*. 2019; Liu *et al*. 2018; Wang *et al*. 2020). Particularly, RNA molecules can form predictable secondary and tertiary structures via self-base pairing (Shapiro *et al*. 2008; Shi *et al*. 2014), allowing finite structural reconfigurations in response to trans-acting molecular stimuli (*e.g*., proteins, metabolites, and other nucleic acids) and environmental cues, which can trigger dynamic switches of their functional states (Bayer and Smolke 2005). This mechanism is fundamental in the genetic and epigenetic regulations of living systems.

**Figure 1:**
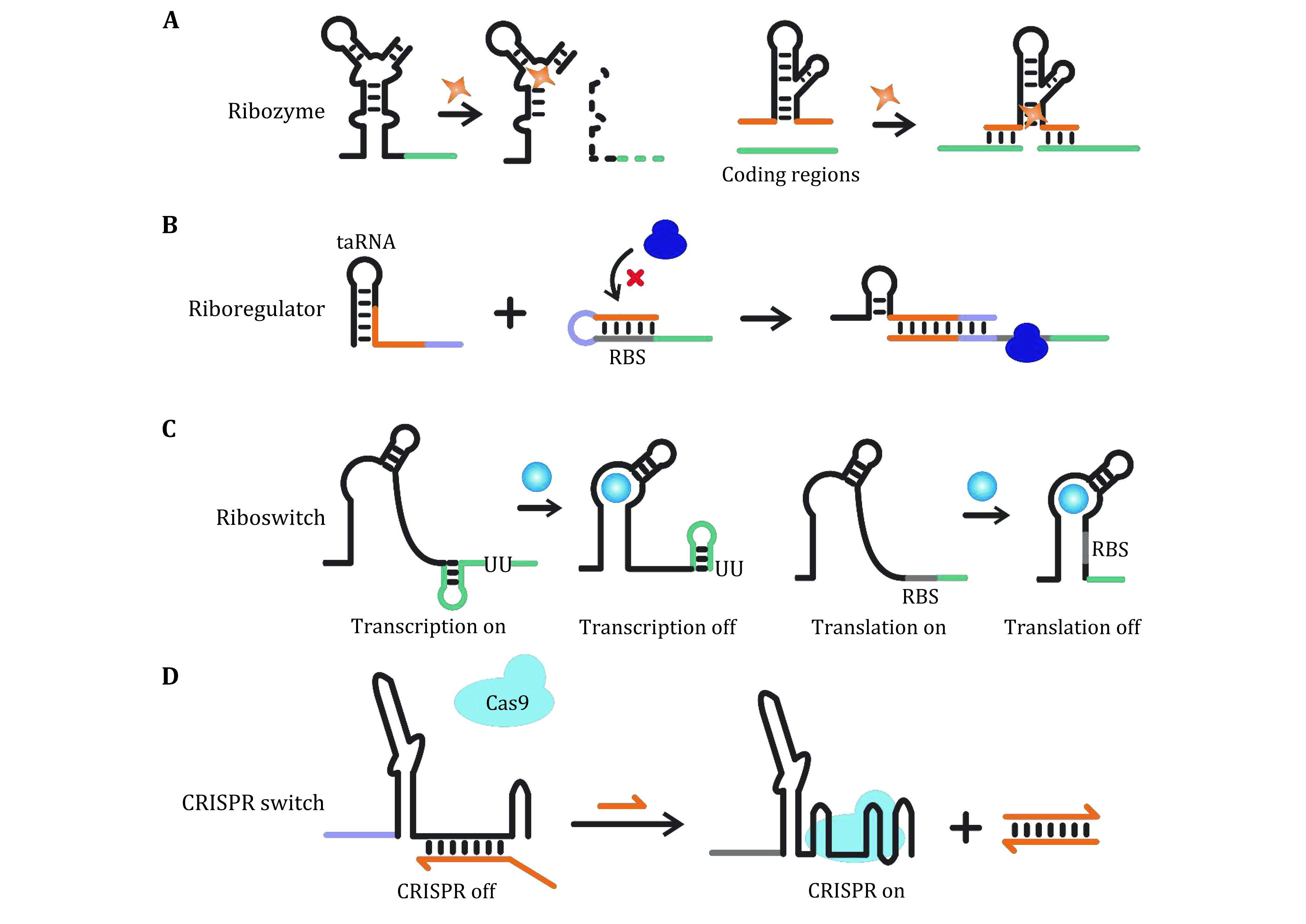
RNA structures with functions that can be switched via structural reconfigurations. **A** Ribozymes and aptamers which are structurally responsive to specific molecules. **B** Riboregulators for trans-acting regulation of gene expression. **C** Riboswitches for cis-acting regulation of gene expression. **D** General schematic of CRISPR switches allowing controllable activities of the CRISPR-Cas system

Recent advances in nucleic acid structural nanotechnology (Fig. 1) have enabled a variety of nanostructures relying on diverse naturally existing or engineered RNA units at the level of secondary and tertiary motifs, including hairpins, kissing loops, kinks (Ohno *et al*. 2011) and three-way junctions. These RNA units can be further assembled into 2D and 3D architectures such as polygons, arrays, and filaments (Grabow and Jaeger 2014). There have been several comprehensive reviews about structural RNA nanotechnology, involving the fundamental aspects of designing, synthesizing, folding and self-assembly of engineered RNA nanostructures (Grabow and Jaeger 2014; Guo 2010; Jasinski *et al*. 2017; Krishnan and Bathe 2012; Qiu *et al*. 2013). Particularly, the development of RNA nanotechnology allows researchers to develop nanomachines based on structurally reconfigurable RNA structures, which provide a powerful toolbox for diverse synthetic biological applications (Ishikawa *et al*. 2013; Li *et al*. 2017; Osada *et al*. 2014).

Engineered RNA structures possess several important advantages in building artificial nanomachines. First, they have excellent programmability, that is, their structures and functionality can be precisely prescribed by their nucleotide sequences. Secondly, RNA motifs are highly modularizable, which can serve as independent and standardized parts allowing nearly arbitrary combinations into complicated systems. Moreover, like their natural counterparts, engineered RNA structures can be synthesized in biological systems. For example, *in vitro* transcription and self-folding of a single-stranded (ss-) RNA into 2D shapes or networks have been demonstrated (Geary *et al*. 2014; Han *et al*. 2017; Yu *et al*. 2015). Recently, the synthesis of RNA nanostructures inside living cells has also been realized via a similar approach (Li *et al*. 2018).

In this review, we focus on recent studies on structurally reconfigurable designer RNA structures. We briefly introduce the basic principles in the design and fabrication of nanomachines driven by RNA structure reconfiguration. We demonstrate some recent applications in gene circuit rewiring, biocomputing, nanofabrication, and smart theranostics based on these RNA nanomachines. We also discuss several limitations and future directions of this field.

## FUNCTIONAL UNITS BASED ON STRUCTURALLY RECONFIGURABLE RNA MOTIFS

In this section, we introduce several basic RNA motifs (Fig. 1), whose functionality can be switched via structural reconfigurations in response to external stimuli. They have been widely exploited for programmable gene regulation in synthetic biology, and can serve as functional modules for the fabrication of nanomachines.

### Ribozymes and RNA aptamers

Ribozymes (or RNA enzymes) are a class of RNA structures with enzymatic activities. They often cooperate with metal cofactors and perform specific phosphodiester bond cleavage and formation at the translational level (Birikh *et al*. 1997; Sigel and Pyle 2007). For example, hammerhead ribozymes are a class of small catalytic RNAs existing in tobacco ringspot viruses, which allow self-cleavage in satellite RNAs in response to magnesium ions signals (Beilstein *et al*. 2015) (Fig. 1A).

Due to recent advances in systematic evolution of ligands by exponential enrichment (SELEX) technology, a variety of artificially selected RNA aptamers and ribozymes have emerged, which can bind the molecules of interest, or show certain catalytic activities when incorporated with given molecules (Darmostuk *et al*. 2015; Ellington and Szostak 1990; Kawazoe *et al*. 2001; Ozer *et al*. 2014). For example, there have been a variety of ribozymes showing activities such as nucleic acid cleavage and ligation. These activities usually depend on their structural configurations, thus are sensitive to external conditions that can alter their structures. On this basis, many ribozyme-based systems have been constructed, which are responsive to environmental factors like metal ions and pH (Lau and Ferre-D'Amare 2016; Li *et al*. 2009).

Recently, a series of RNA aptamers that can mimic fluorescent proteins (fluorescence light-up aptamers) have attracted much interest. These RNA aptamers light up when binding to corresponding fluorophores (Neubacher and Hennig 2019; Song *et al*. 2014), and are named after vegetables according to their fluorescence colors (*e.g*., spinach, broccoli, corn, and mango). Like their protein counterparts, these RNA aptamers can be fused to other RNA molecules of interest, thus are suitable for RNA imaging and detection in live cells. These fluorescence aptamers can be further integrated with other structurally reconfigurable aptamers, enabling switchable fluorescence in response to the molecules of interest (Jepsen *et al*. 2018; Paige *et al*. 2012; Pothoulakis *et al*. 2014). Recently, an aptamer-initiated fluorescence complementation (AiFC) method has been developed for RNA imaging (Wang *et al*. 2018b). In this method, a broccoli motif is split into two fragments unable to emit fluorescence, which can recover the intact form and light up the fluorescence upon binding to the target RNA, enabling high-contrast and real-time imaging of endogenous RNAs. On the other hand, these fluorescent RNA aptamers can be utilized in other synthetic nucleic acid nanodevices as output signals (Chakraborty *et al*. 2016; Jiao *et al*. 2020).

### Riboswitches

In nature, riboswitches refer to cis-acting RNA motifs located in the regulatory regions (*e.g*. 5' or 3' untranslated regions, UTRs) of mRNAs (Fig. 1C). They can be structurally reconfigured upon binding with specific small molecules (Bailor *et al*. 2010), and then sequester or expose key regulatory sites (*e.g*., ribosome binding site, RBS), allowing switches of translation initiation or transcription termination (Batey *et al*. 2004; Nudler and Mironov 2004). Inspired by naturally existing riboswitches, artificially designed RNA motifs allowing responsive structural reconfigurations have been designed, which can serve as binary switches enabling transduction of input signals into various outputs, thus have been widely used in synthetic biology (Dwidar *et al*. 2019; Ogawa and Maeda 2008).

#### Riboswitches at the transcriptional level

**Figure 2:**
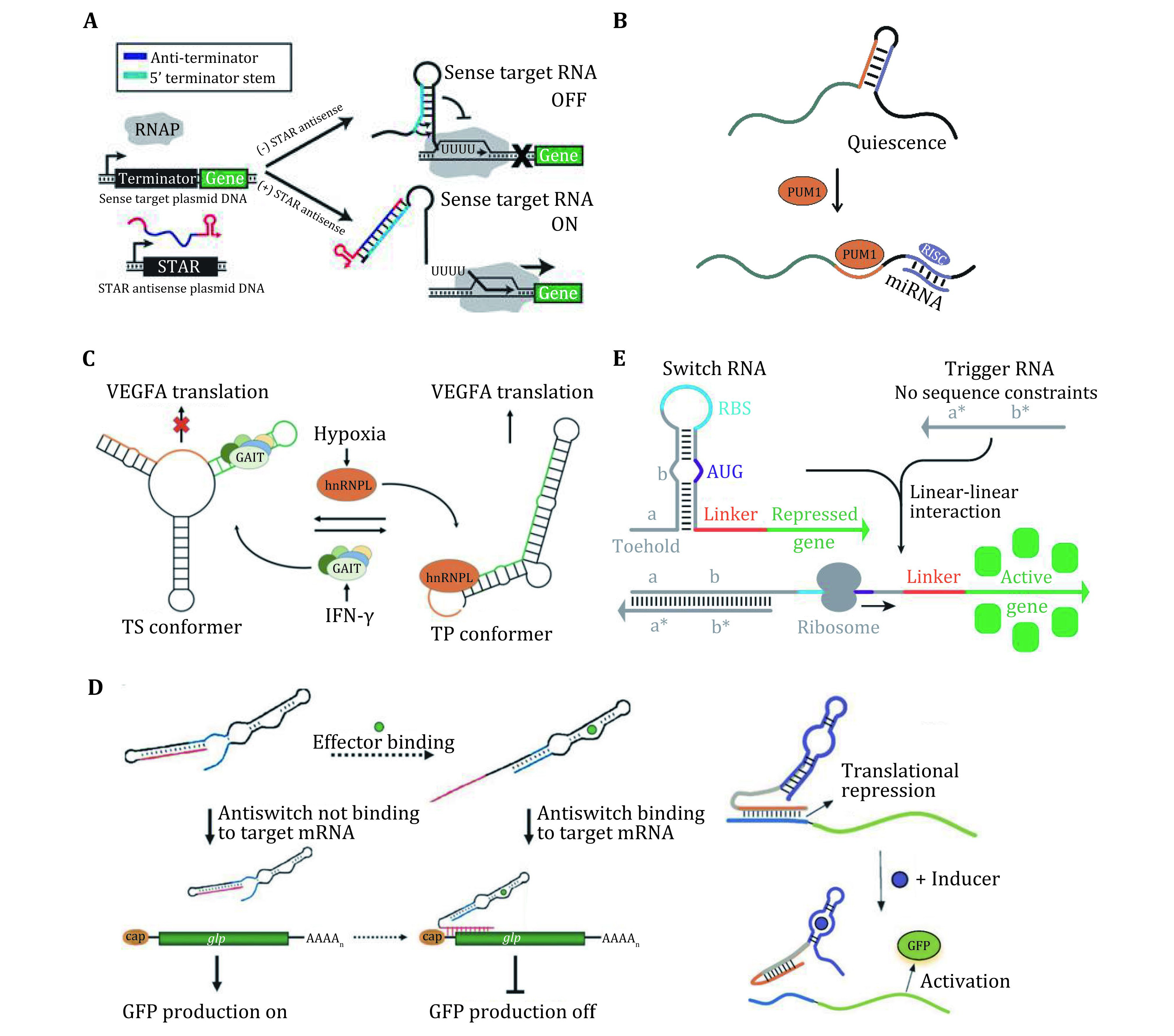
**A** Schematic of an anti-terminator STAR. This system inverts the structural configuration of the attenuator through the addition of an anti-terminator sequence. Adapted from Meyer *et al*. (2016). **B** Pumilio protein-mediated mRNA secondary structural switch controls the accessibility of microRNA-binding sites and regulates the protein expression. RISC, RNA-induced silencing complex. **C** Translation control of gene expression through a dual protein-dependent RNA secondary structural switch that responds to interferon-γ (IFN-γ). Adapted from Liu *et al*. (2016a). **D** Engineered riboregulators responsive to small molecules, which control the gene translation in a trans-acting manner. Adapted from Agapakis and Silver (2009) and Munzar *et al*. (2019). **E** Toehold switch system comprising a switch RNA for repressing translation, and a trigger RNA with arbitrary sequence which can reconfigure the switch RNA via a toehold-mediated linear-linear interaction. Adapted from Green *et al*. (2014)

Many riboswitches regulate gene expression at the transcriptional level by producing transcription-terminating helices. For example, a series of synthetic small transcription activating RNAs (STARs) have been constructed to disrupt the formation of an intrinsic transcription terminator placed upstream of a gene in *Escherichia coli* (*E. coli*) (Chappell *et al*. 2015; Meyer *et al*. 2016) (Fig. 2A). Moreover, multiple orthogonal STARs can be built and assembled in tandem in one RNA molecule, enabling RNA-only transcriptional logic gates.

#### Riboswitches at the post-transcriptional level

A variety of riboswitches have been found involved in regulating post-transcriptional processing (Abbink *et al*. 2005; Winkler and Breaker 2005). For example, splicing, gene silencing by microRNA (miRNA), and RNA editing. Although the detailed mechanisms behind these systems are poorly understood, these riboswitches enable controllable exposure, occlusion, or modulation of the processing sites to regulate post-transcriptional processes. For example, a riboswitch (Kedde *et al*. 2010) has recently been identified in the 3’-UTR of p27 mRNA. This riboswitch is a secondary structure that simultaneously sequesters two regions: one is a microRNA (miRNA) binding site, the other is a pumilio-recognition element (PRE) which can bind a pumilio RNA-binding protein (PUM1). The binding of PUM1 and PRE can trigger a structural reconfiguration of the riboswitch and expose the miRNA binding site, leading to miRNA-induced gene silencing (Fig. 2B).

#### Riboswitches at the translational level

Riboswitches functioning at the translational level have also been intensively exploited category of switches in synthetic biology. They usually rely on structurally reconfigurable motifs at the 5' or 3' UTR, which can sequester or expose the RBS in response to specific stimuli, thus switch the translational activity of the downstream coding regions (Isaacs *et al*. 2006). For example, a riboswitch is responsive to thiamine or its pyrophosphate derivative without the need for protein cofactors (Winkler *et al*. 2002). The binding of the effector can trigger a reconfiguration of the riboswitch, which sequesters the RBS and leads to a suppression of gene expression in *E. coli*.

Recently, a protein-dependent riboswitch has been identified in the 3ʹ UTR of VEGFA mRNA in myeloid cells that regulates translation of VEGFA in response to proteins associated with two disparate stress stimuli (Fig. 2C). The interferon-γ (IFN-γ)-activated inhibitor of translation (GAIT)-complex binds a structural GAIT element within a family of inflammatory mRNAs and silences their translation by promoting the formation of a translational-silencing (TS) conformer (Ray *et al*. 2009). During oxidative stress, the heterogeneous nuclear ribonucleoprotein L (hnRNP L) overrides GAIT silencing by triggering a secondary structural RNA switch to a translation-permissive (TP) conformer, in which the GAIT element is occluded. The RNA alternates between two mutually exclusive conformers in response to the binding of the GAIT complex or hnRNP L, thereby functioning as an AND NOT Boolean logic-gate switch in which the presence of one protein, but not the other, yields an output of gene repression.

### Riboregulators

In contrast to riboswitches that are *cis*-acting motifs, riboregulators usually refer to *trans*-acting RNAs (or antisense RNAs) that can bind and reconfigure the regulatory sites (*e.g*., riboswitches) of other mRNAs, leading to post-transcriptional/translational regulations of the latter (Fig. 1B). This class of RNA structures has also been widely employed in synthetic biology (Ueno *et al*. 2017, 2018; Wang *et al*. 2018a).

For example, Bayer *et al*. designed a set of riboregulators called "antiswitches" (Fig. 2D) (Bayer and Smolke 2005), which can function in *Saccharomyces cerevisiae*. The riboregulators contain an aptamer domain, whose structure can be reconfigured in response to specific ligands, leading to switchable sequester/exposure of the antisense domain, and thus switchable binding/unbinding of the target mRNAs. By the rational design, the translation of the target mRNAs can be activated (by the on-antiswitch) or repressed (by the off-antiswitch) in the presence of the ligands, which demonstrates the capability of these riboregulators for bi-directional modulation.

In these earlier strategies, the riboregulators usually rely on U-turn loop structures to drive loop-linear interactions between RNAs (Callura *et al*. 2012; Daniel *et al*. 2013). The riboregulators need to contain sequences antisense to the riboswitches (including the RBS), which largely limits their design freedom. Lately, Yin and coworkers (Green *et al*. 2014) established a new set of riboswitches (referred to as switch RNA) and riboregulators (referred to as trigger RNA), dubbed "Toehold Switch" (Fig. 2E). In this system, a "toehold" sequence is introduced on the switch RNA at the 5' UTR, thus the trigger RNA can reconfigure the switch RNA via a toehold-mediated linear–linear interaction. This strategy allows us to design riboregulators with arbitrary sequences. Moreover, the toehold switches provide a high dynamic range (>400) as well as a high level of orthogonality. They can be applied in the bacterial genome to regulate endogenous genes.

## INTEGRATION OF RNA UNITS INTO NANOMACHINES

Till now, a variety of reconfigurable RNA units possessing diverse functions have been established, which encourage researchers to integrate them into smart nanomachines or nanorobots. This pursuit requires that the functionality of the RNA units should be well maintained in the integrated systems, with undesired crosstalk minimized. Moreover, non-RNA molecules, especially proteins, can be integrated and cooperate with the RNA units, enabling functions unable to be provided by pure RNAs. Here we discuss two representative approaches for the integration.

### Assembly of RNA units with RNA scaffolds

Owing to the excellent modularity of RNA motifs and the advances of RNA nanotechnology, diverse functional RNA units can be nearly arbitrarily assembled into complex systems with the assistance of RNA scaffolds, which enable multifunctional nanodevices and smart nanomachines (Khaled *et al*. 2005; Shu *et al*. 2011). Moreover, the structural stability of the functional RNA units can be largely enhanced.

**Figure 3:**
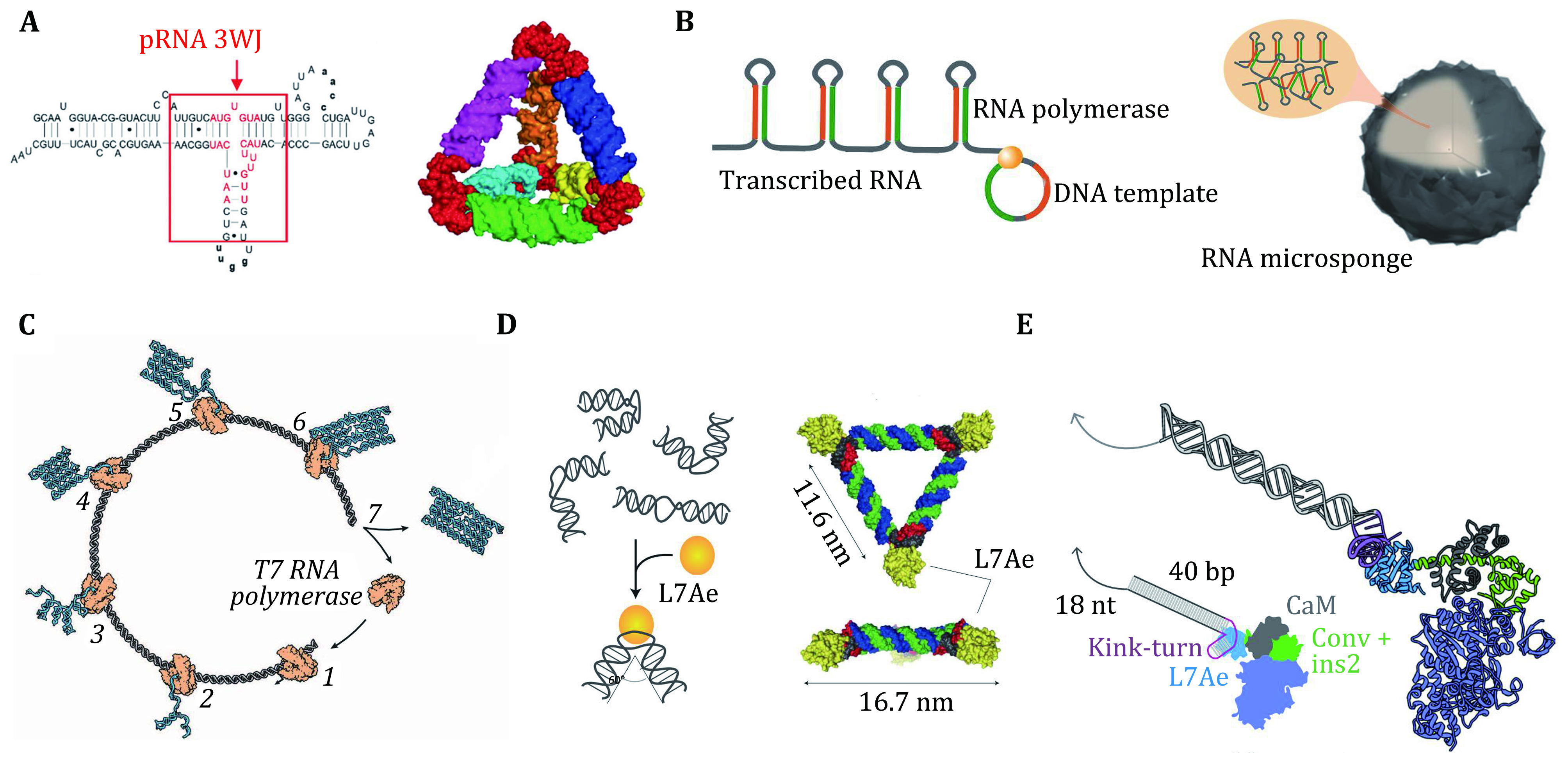
**A** An RNA tetrahedron assembled by the three-way junction (3WJ) motifs from pRNA. Adapted from Li *et al*. (2016). **B** Rolling circle transcription (RCT) for the self-assembly of RNA-microsponges. Adapted from Yuan *et al*. (2019). **C** RNA origami structure generated via a cotranscriptional folding pathway. The T7 RNA polymerase binds to the template DNA (step 1) and the RNA folds as it is being synthesized (steps 2 to 7). Adapted from Geary *et al*. (2014). **D** Molecular design of a triangular RNP assembled based on protein-RNA interactions. Adapted from Durbin *et al*. (2019). **E** Design and characterization of an engineered myosin with an RNA lever arm. Adapted from Saper and Hess (2020)

For example, Guo's group (Li *et al*. 2016; Shu *et al*. 2015) utilized three-way junctions (derived from the packaging RNA, or pRNA, in a bacteriophage) and four-way junctions as building blocks to build multimeric RNA nanostructures (Fig. 3A). These structures as scaffolds can be used to integrate multiple ribozymes and aptamers into multifunctional nanodevices (Afonin *et al*. 2014; Delebecque *et al*. 2011; Hao *et al*. 2014). Rolling circle transcription has also been used to synthesize RNA nanoparticles carrying periodically arranged functional motifs (Geary *et al*. 2014; Jang *et al*. 2015; Lee *et al*. 2012) (Fig. 3B). More recently, RNA origami structures, folded by a long ssRNA (up to ~6000 nt) via transcription have shown promise in serving as scaffolds to assemble nanomachines (Geary *et al*. 2014; Grabow and Jaeger 2014; Han *et al*. 2017; Li *et al*. 2018) (Fig. 3C). Theoretically, they allow spatial arrangement of the functional RNA motifs in nanometer precision, and more importantly, can be synthesized in living systems.

### Integration with proteins

Many natural nanomachines in living organisms are in form of ribonucleoproteins (RNPs, or RNA-protein complexes, such as ribosomes, telomerase, and small nuclear ribonucleoproteins) (Greider and Blackburn 1987; Maniatis and Reed 1987). Inspired by them, natural RNA motifs or artificially selected RNA aptamers that can bind specified proteins have been harnessed to assemble hybrid nanomachines akin to natural RNPs.

For example, the kink-turn (K-turn) RNA motifs can interact with K-turn binding protein L7Ae and conduct structural reconfigurations, which can be utilized to build RNP nanomachines that can transform in response to L7Ae (Ohno *et al*. 2011; Osada *et al*. 2014) (Fig. 3D). Recently, a new type of myosin motors incorporating RNA lever arms has been constructed based on the binding of K-turn and L7Ae (Omabegho *et al*. 2017) (Fig. 3E). The structural changes in the protein motor domain are amplified and redirected by the RNA structures. The speed and direction of motor motion can be dynamically controlled by the programmable transitions of RNA lever arm structure in response to strand-displacement reactions. In multimeric assemblies, the motors can walk processively along actin filaments. Moreover, this system can implement orthogonal responses of RNA variants to specific oligonucleotides.

### Integration with CRISPR-Cas system

Clustered regularly interspaced short palindromic repeats (CRISPR)-CRISPR associated protein (Cas) system can be regarded as a natural ribonucleoprotein machine for adaptive immunity in microorganisms (Gasiunas *et al*. 2012), which has been intensively exploited as a powerful gene-editing tool. For example, a typical CRISPR-Cas9 system relies on guide RNAs (gRNAs) which bind the Cas9 endonuclease and guide the cleavage of target double-stranded (ds-) DNA in a sequence-specific manner. Herein, by engineering the structure of guide RNAs, their ability of Cas binding and/or targeting sequence recognition can be altered (Fig. 1D), enabling nanomachines with switchable targeting and cleavage activity (Jin *et al*. 2019; Liu *et al*. 2016b; Wang *et al*. 2019; Zalatan *et al*. 2015).

**Figure 4:**
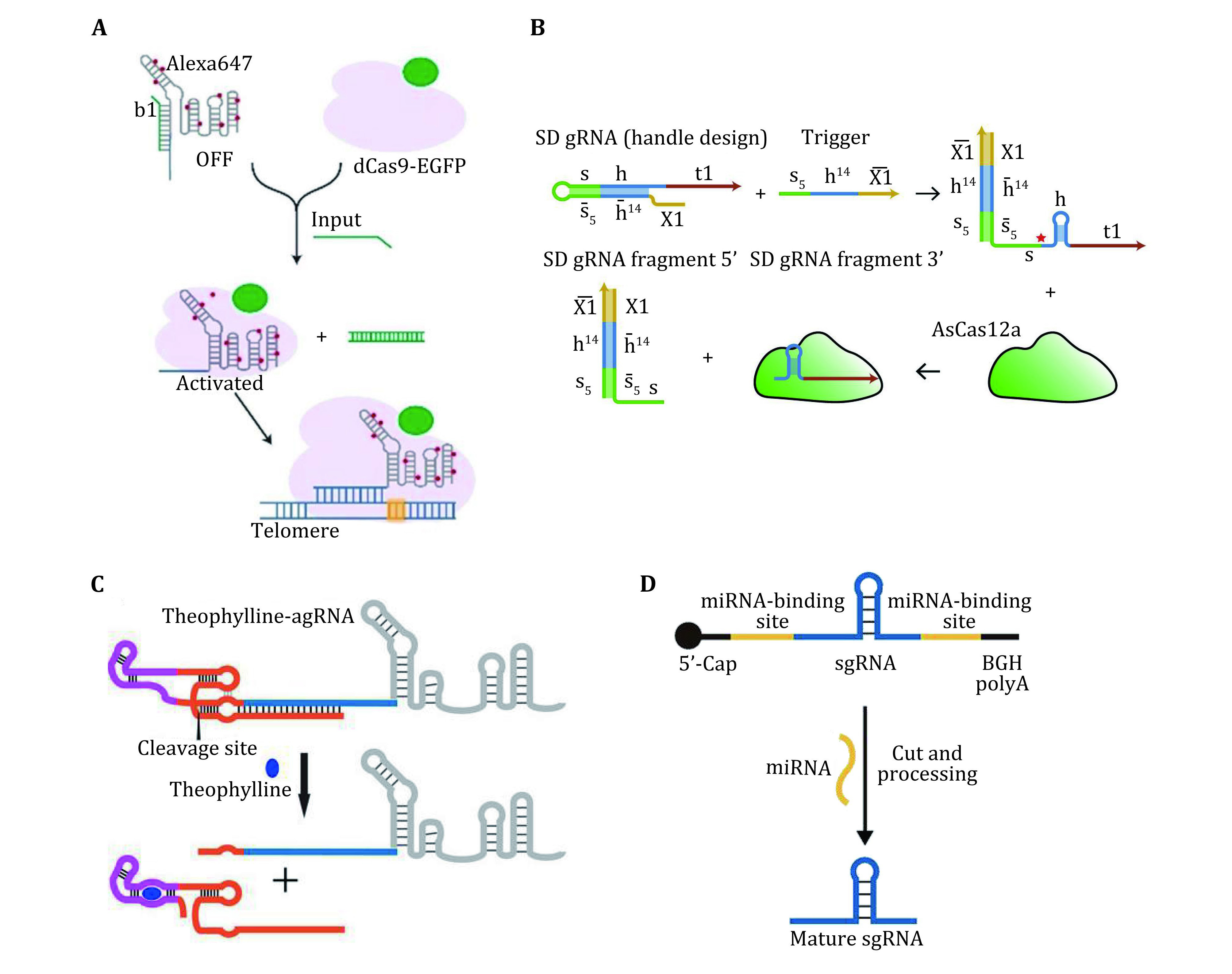
**A** Design of a trigger YES gate for the activation of telomere imaging. Adapted from Hao *et al*. (2020). **B** Principle of strand displacement switchable gRNAs. RNA trigger binds the SD gRNA, thereby restoring the gRNA handle. Binding of Cas12a leads to cleavage of the gRNA, and creates an active Cas12a-gRNA complex. Adapted from Oesinghaus and Simme (2019). **C** Schematic of the activation of theophylline aptazymes modified sgRNA in the presence of theophylline. Adapted from Tang *et al*. (2017). **D** Schematic representation of the activation of sgRNA by miRNA triggered cleavage. Adapted from Zhu *et al*. (2020)

For example, our group has developed a switchable CRISPR-Cas9 system (Hao *et al*. 2020), which can respond to multiple nucleic acid inputs and reconfigure the structure of the gRNA through toehold-switch-mediated strand displacement (Fig. 4A). This system can implement orthogonal suppression and activation of the Cas9 targeting ability in response to specific DNA inputs. The combination of toehold switches enables diverse intracellular Cas9 activation programs with simultaneous and orthogonal responses.

Similarly, Oesinghaus *et al*. has reported a programmable activation strategy for the Cas12a system (Oesinghaus and Simme 2019). This system relies on designed strand displacement gRNAs (SD gRNAs) that can be specifically activated by trigger RNAs. This system enables logical transcriptional control of gene expression in *E. coli* (Fig. 4B).

By augmenting guide RNAs with self-cleaving ribozyme motifs (aptazymes) responsive to small molecules, Tang *et al*. (Tang *et al*. 2017) developed a CRISPR-Cas9 system allowing small molecule-controlled genome editing and small molecule-dependent transcriptional activation in mammalian cells (Fig. 4C).

In another example, Wang *et al*. designed an inactive sgRNA precursor (pre-sgRNA) (Wang *et al*. 2019), which could be cleaved by Argonaute (AGO) proteins upon binding with specific microRNAs (miRNAs), releasing functional sgRNAs generating that could guide the Cas9 to regulate the expression of reporter genes (Fig. 4D). By designing the targeting and miRNA binding sequence of pre-sgRNA, this system can be applied to control the expression of specified endogenous genes or mutate specific DNA bases in response to cell type-specific miRNAs.

## TARGETED APPLICATIONS

Given the advances of nanomachines empowered by structurally reconfigurable RNA structures, their potential for biological and biomedical applications has also attracted broad interest. Here we summarize recent progress in their applications in cellular logic computation, diagnosis, and therapy.

### Cellular logic computation

**Figure 5:**
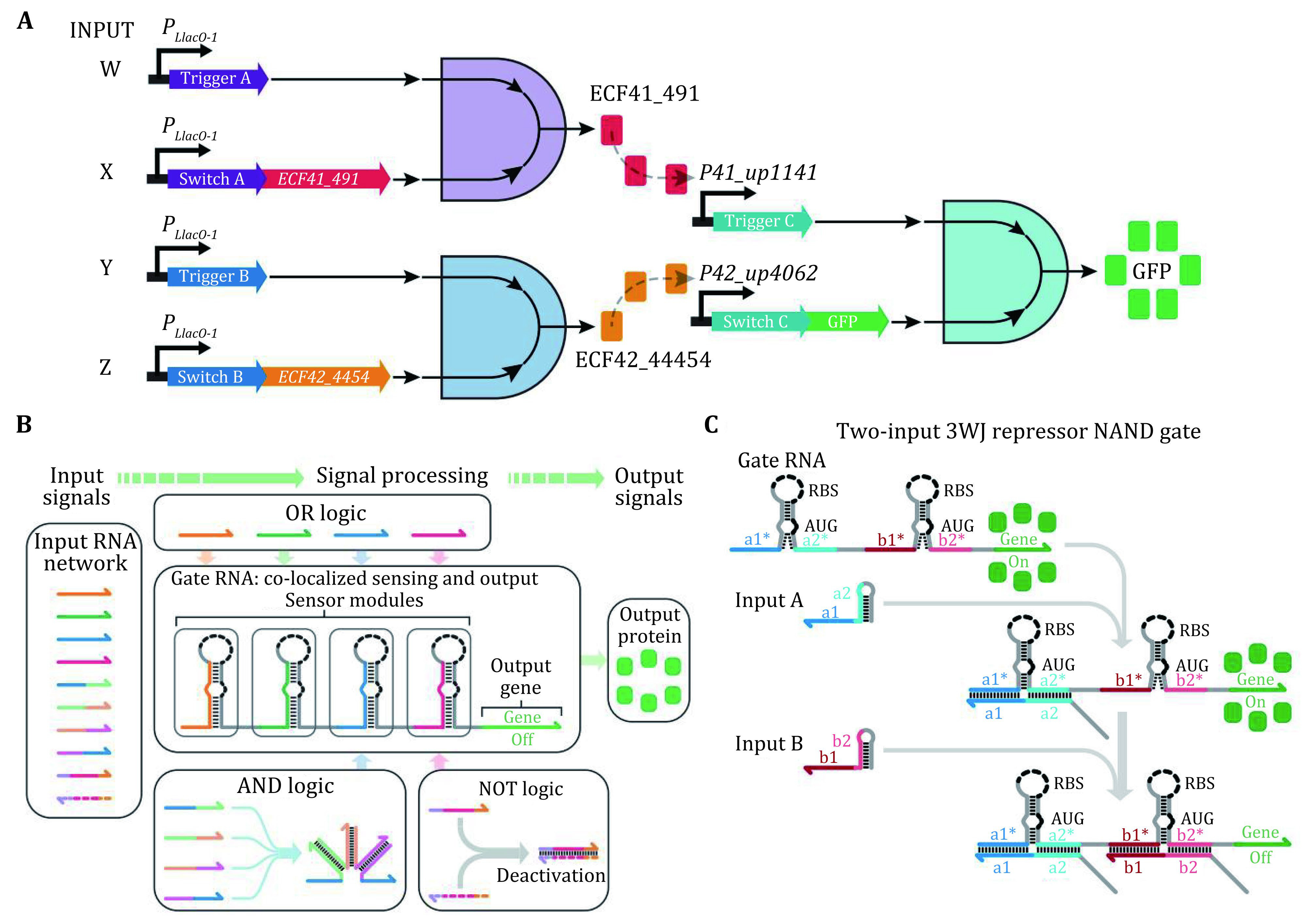
**A** Design schematic for the four-input AND circuit by three toehold switches, two orthogonal transcription factors, and a GFP reporter. Adapted from Green *et al*. (2014). **B** Ribocomputing system using RNA molecules as input signals and fluorescent protein as the output signal. Signal processing is carried out by a gate RNA that co-localizes sensing and output modules. Adapted from Simmel *et al*. (2019). **C** Design of a 3WJ repressor NAND gate. In the gate RNA, two switch modules are inserted in-frame and upstream of the reporter gene and both input RNAs must bind to the gate to prevent gene expression. Adapted from Kim *et al*. (2019)

A cell is in principle an elaborate natural biocomputer that collects physical/chemical information from the environment, performs calculations via signal pathways or molecular circuitry, and uses this data to perform actions (Regev and Shapiro 2002). So far, most designed biological circuits are highly case specific, making them difficult to adapt and transplant from one organism to another. Hence, it is highly desirable to develop a more general synthetic circuitry scheme. Toehold-based RNA switches provide a powerful tool to realize such organism independent circuitry given their ubiquity in all forms of life and their predictable base pairing interactions. Yin and coworkers (Green *et al*. 2014) exploited orthogonal toehold switches to regulate 12 genes independently and to construct a circuit that can conduct four-input AND logic calculation (Fig. 5A). However, it remains challenging to scale up these circuits owing to the limited number of designable, orthogonal components. Later, they reported a "ribocomputing" system composed of *de-novo*-designed RNA parts, which can perform complex logic calculations in living cells with high dynamic range (Green *et al*. 2017). This ribocomputing system can be scaled up to calculate four-input AND, six-input OR, and a complex 12-input expression (Fig. 5B). On this basis, they introduced a new type of *de-novo* synthetic riboregulators, termed three-way junction (3WJ) repressors (Kim *et al*. 2019), which can help detect transcripts with nearly arbitrary sequences, repress gene expression by up to 300-fold and yield orthogonal sets of up to 15 devices. The modular repressors can be integrated into biological circuits that execute universal NAND and NOR logic and evaluate the four-input expression in *E. coli* (Fig. 5C).

### Diagnosis

The increasing ability of synthetic biologists to repurpose and engineer reconfigurable RNA components for nanomachines has led to new opportunities for molecular diagnostics, especially for nucleic acid detection (Chan and Ng 2015; Khan 2006; Slomovic *et al*. 2015). The most prominent advantage might be that they can work under ambient conditions, *e.g*., detect nucleic acids under isothermal conditions without the need for thermal cycling equipment. Thus, they as easy-to-use platforms have the potential for point-of-care diagnosis (Thavarajah *et al*. 2020).

For example, Collins and coworkers developed toehold-based RNA switches as programmable RNA sensors (Green *et al*. 2014), which can be rationally designed to bind and sense virtually any RNA sequence. A freeze-dried, paper-based, cell-free protein expression platform, allows for the deployment of these toehold switch sensors outside of a research laboratory by providing a sterile and abiotic method for the storage and distribution of genetic circuits at room temperature (Pardee *et al*. 2014). They combined these technologies to create a platform for rapidly and inexpensively developing and deploying diagnostic sensors.

**Figure 6:**
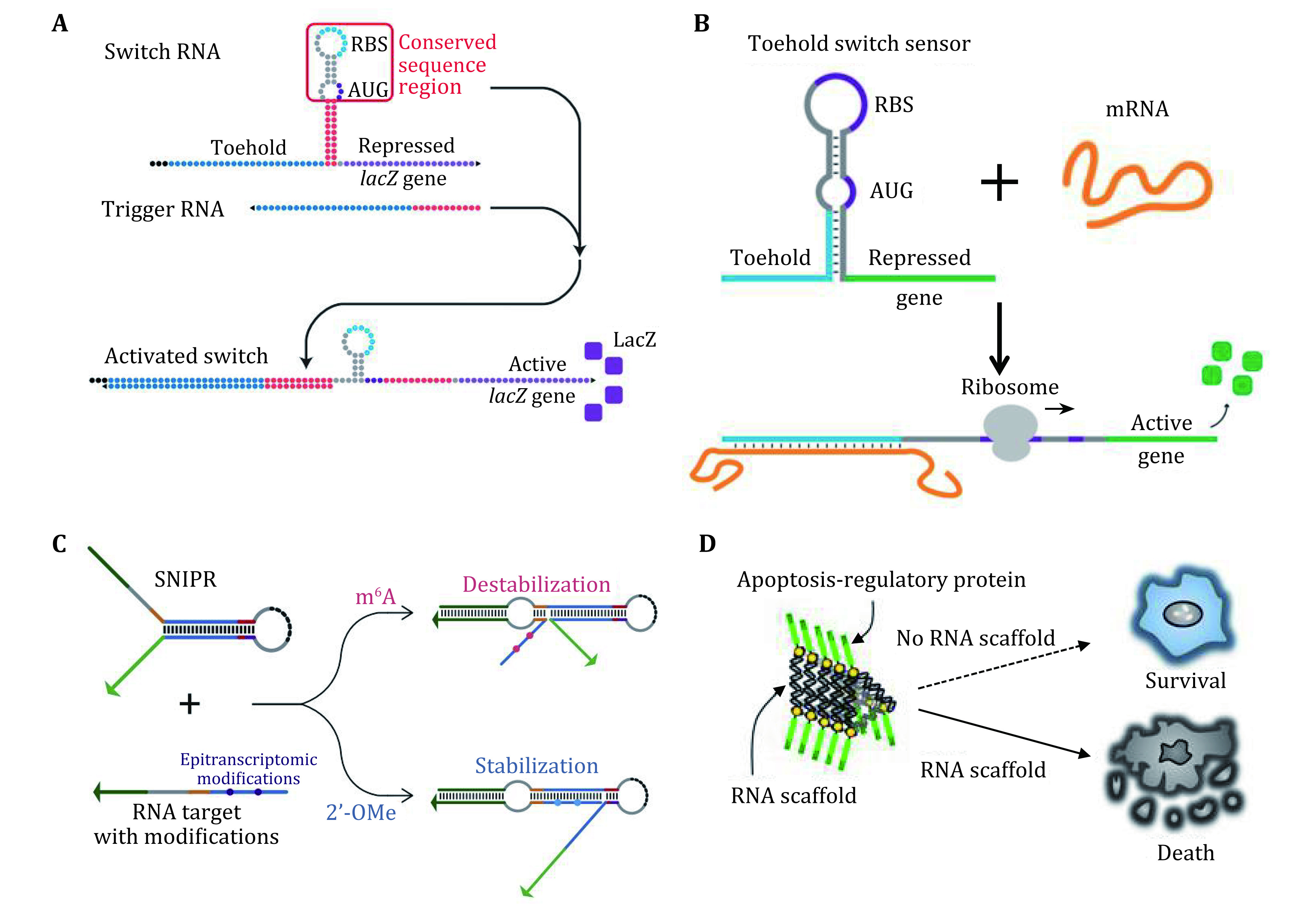
**A** Zika virus toehold switch sensor. The target RNA from the Zika virus can trigger the reconfiguration of the switch RNA and activate the expression of the reporter gene *lacZ*. Adapted from Pardee *et al*. (2016). **B** Schematic of toxin mRNA toehold switch sensor function. Adapted from Takahashi *et al*. (2018). **C** Principle of SNIPR for detection of epitranscriptomic marks, which can identify epigenetically modified nucleobases in target RNAs. Adapted from Hong *et al*. (2020). **D** An RNP nanomachine for inducing tumor cell apoptosis by oligomerization of apoptosis regulatory proteins. Adapted from Shibata *et al*. (2017)

A later report examined the diagnostic capability of a toehold switch in which the trigger was Zika virus RNA, and the mRNA under control encoded an enzymatic "reporter" protein (Pardee *et al*. 2016). These paper based switches could reliably detect the Zika virus RNA with great sensitivity (Fig. 6A). Furthermore, the switches worked even after long term storage at ambient temperature. Although designed for use in detecting bacteria, paper based toehold switches also can quantify mRNA and validate the platform on clinical stool samples by comparison to RT-qPCR. They further highlight the potential clinical utility of the platform by showing that it can be used to rapidly and inexpensively detect toxin mRNA in the diagnosis of *Clostridium difficile* infections (Fig. 6B) (Takahashi *et al*. 2018). Alexander A. Green developed single-nucleotide-specific programmable riboregulators (SNIPRs) which provide over 100-fold differences in gene expression in response to target RNAs differing by a single nucleotide in *E. coli* and resolve single epitranscriptomic marks *in vitro* (Hong *et al*. 2020). By exploiting the programmable SNIPR design, integrating SNIPRs with portable paper-based cell-free reactions enables convenient isothermal detection of cancer-associated mutations from clinical samples (Fig. 6C).

Nanomachines based on CRISPR-Cas systems have also been harnessed in molecular diagnosis. For example, the miRNA-induced Cas9-sgRNA activating system developed by Wang *et al*. (Wang *et al*. 2019) can be used for high-fidelity sensing of miRNA activity at cellular levels and monitoring of differentiation status of stem cells. CRISPR-Cas12 system presents indiscriminate ssDNA cleavage activity upon RNA-guided DNA binding (Chen *et al*. 2018). Thus, an ssDNA labeled with both a fluorescence dye and a quencher can be used as a reporter, which is cleaved by Cas12 and generates a fluorescent signal in the presence of the target DNA. This principle recently has been utilized for target-specific isothermal signal amplification in nucleic acid assays (*e.g*., detection of SARS-CoV-2 viral nucleic acids) (Broughton *et al*. 2020; Chen *et al*. 2018). Similarly, CRISPR-Cas13 mediates collateral cleavage of RNAs upon RNA-guided RNA recognition, which has also been harnessed for detection of pathogenic nucleic acids (*e.g*., nucleic acids from Zika virus, dengue virus, or other pathogenic bacteria) (Gootenberg *et al*. 2017; Myhrvold *et al*. 2018).

### Therapeutics

Nanomachines based on reconfigurable RNA structures have also shown potential in biomolecular therapeutics. For instance, RNA nanostructures can be used to incorporate RNA aptamers targeting tumor markers (*e.g*., human epidermal growth factor receptor, or EGFR) and therapeutic RNAs such as small interfering RNAs (siRNAs) and anti-miRNAs, enabling cancer-targeted therapeutics (Jasinski *et al*. 2017).

Controllable CRISPR-Cas9 nanomachines have also shown potential in therapeutics. Recently, a CRISPR-Cas9 system incorporating riboswitches has been used to simultaneously regulate multiple genes in an oncogenic pathway, allowing reprogramming of the fate of cancer cells (Liu *et al*. 2016b), which has implications for cancer therapeutics.

More recently, a type of protein-driven RNA nanostructured devices has been constructed, which can be activated *in vitro* by RNA-binding-protein-inducible structural change, which can control the assembly and oligomerization of apoptosis-regulatory proteins via specific RNA–protein interactions, leading to selective killing of target cells (Shibata *et al*. 2017) (Fig. 6D).

## CONCLUSIONS AND PERSPECTIVES

In summary, we have reviewed the development of structurally reconfigurable RNA structures and nanomachines empowered by them. These studies show that by rationally programming RNA sequences, RNA structures with tailored thermodynamic and structural properties can be obtained, along with prescribed functionality (Chakraborty *et al*. 2014). Especially, reconfigurable RNA modules, which possess fast response kinetics to biological and environmental stimuli, can be employed in nanomachines to sense signals including nucleic acids, proteins metabolites, or other small molecules, and carry out specific tasks correspondingly.

Despite such progress, there remain challenges for the transition of the engineered RNA nanomachines into practical theranostic applications. First, compared to many inorganic or polymeric materials, RNA molecules are highly degradable due to ubiquitous RNases in practical environments, leading to very short lifetimes of the nanomachines and constrained application scenarios. Thus, *in-situ* fabrication of RNA nanomachines in living cells is highly desired, which allows them to be continuously produced utilizing the transcriptional machinery of the hosts. This approach also ensures that they are recycled rapidly after use, thus minimizes the biosafety risks resulted from long-term accumulation. On the other hand, the stability of RNA structures can be largely improved by introducing chemical modifications (Jasinski *et al*. 2017) or non-natural nucleotides, structural optimization (Guo *et al*. 2020; Khisamutdinov *et al*. 2014), and incorporation with protective materials.

Second, the complexity of *de-novo* designed RNA nanostructures is so far quite limited compared to DNA nanostructures (*e.g*., DNA origami structures) (Geary *et al*. 2014; Grabow and Jaeger 2014; Han *et al*. 2017; Li *et al*. 2018). For nucleic acid self-assembly, the target structures are designed to be thermodynamically stable, but often not kinetically favored. The folding and assembly of complex RNA structures are more likely to be kinetically trapped, resulting in incorrect products. This could be attributed to that the base-pairing of RNA is stronger than that of DNA. Therefore, it is highly desired to develop easy-to-use designing principles and tools for RNA nanostructures, which can well avoid kinetic traps and allow more complex RNA nanostructures (Geary *et al*. 2014; Grabow and Jaeger 2014; Han *et al*. 2017; Li *et al*. 2018).

The combination with artificial evolution strategies (*e.g*., SELEX) may help construct more complicated RNA systems with optimized performances. However, the efficiency of traditional evolution techniques is poor. It often takes years to find a new RNA structure with the desired functionality. Therefore, there is a need to develop high-throughput yet high-speed selection strategies for evolving input-output responses and dynamic functions.

Taken together, we envision that in near future, the development of cutting-edge technologies in diverse fields would largely enrich the catalog of designer RNA structures, which enable novel nanomachines with functions that are robust, scalable, and applicable to a wide range of living organisms.

## Conflict of interest

Kai Jiao, Yaya Hao, Fei Wang, Lihua Wang, Chunhai Fan and Jiang Li declare that they have no conflict of interest.
